# A Three-Dimensional Micromixer Using Oblique Embedded Ridges

**DOI:** 10.3390/mi12070806

**Published:** 2021-07-08

**Authors:** Lin Li, Qingde Chen, Guodong Sui, Jing Qian, Chi-Tay Tsai, Xunjia Cheng, Wenwen Jing

**Affiliations:** 1 Department of Ocean & Mechanical Engineering, Florida Atlantic University, 777 Glades Road, Boca Raton, FL 33431, USA; xiaoshu1983383@gmail.com (L.L.); qchen4@fau.edu (Q.C.); 2 Department of Environmental Science and Engineering, Fudan University, Shanghai 200032, China; gsui@fudan.edu.cn; 3 College of the Environment & Ecology, Xiamen University, Xiamen 361102, China; silence2121@163.com; 4 Department of Medical Microbiology and Parasitology, School of Basic Medical Sciences, Fudan University, Shanghai 200032, China; 5 Key Laboratory of Medical Molecular Virology (MOE/NHC/CAMS), School of Basic Medical Sciences, Shanghai Medical College, Fudan University, Shanghai 200032, China

**Keywords:** micromixer, chaotic advection, vortex transverse flow, complete mixing

## Abstract

A micromixer is one of the most significant components in a microfluidic system. A three-dimensional micromixer was developed with advantages of high efficiency, simple fabrication, easy integration, and ease of mass production. The designed principle is based on the concepts of splitting–recombination and chaotic advection. A numerical model of this micromixer was established to characterize the mixing performance for different parameters. A critical Reynolds number (Re) was obtained from the simulation results. When the Re number is smaller than the critical value, the fluid mixing is mainly dependent on the mechanism of splitting–recombination, therefore, the length of the channel capable of complete mixing (complete mixing length) increases as the Re number increases. When the Re number is larger than the critical value, the fluid mixing is dominated by chaotic advection, and the complete mixing length decreases as the Re number increases. For normal fluids, a complete mixing length of 500 µm can be achieved at a very small Re number of 0.007 and increases to 2400 µm as the Re number increases to the critical value of 4.7. As the Re number keep increasing and passes the critical Re number, the complete mixing length continues to descend to 650 µm at the Re number of 66.7. For hard-to-mix fluids (generally referring to fluids with high viscosity and low diffusion coefficient, which are difficult to mix), even though no evidence of strong chaotic advection is presented in the simulation, the micromixer can still achieve a complete mixing length of 2550 µm. The mixing performance of the micromixer was also verified by experiments. The experimental results showed a consistent trend with the numerical simulation results, which both climb upward when the Re number is around 0.007 (flow rate of 0.03 μm/min) to around 10 (flow rate of 50 μm/min), then descend when the Re number is around 13.3 (flow rate of 60 µm/min).

## 1. Introduction

A microfluidic system, which is also named a lab-on-a-chip (LOC), can perform analytical chemistry [1,2,3], biomedical diagnosis [4,5,6,7], chemical synthesis [8,9], clinical diagnostics [10,11,12,13], biological analysis [14,15,16], etc. in one small chip. It is more advantageous compared to traditional methods, since it can consume very small amounts of samples to conduct the experiment with faster reactions. In various kinds of operational and functional microfluidic chips, the micromixer is an essential component in integrated microfluidic systems and is involved in the sample preparation stage of a chemical analysis [17] and chemical or biological reactions. Therefore, the vital function of the micromixer is using the shortest microchannel length and less time to achieve high mixing efficiency. However, due to its small size and slow flow rate, the fluid is laminar flow with a small Reynolds number (Re), which is defined as the ratio of inertial force to the viscous forces in a fluid and follows the formula Re=ρuLμ, where ρ is the density of the fluid, u is the flow speed, *L* is the characteristic linear dimension, and μ is the dynamic viscosity of the fluid. The flow at a low Re number is usually laminar flow, while the flow at a high Re number is usually turbulent. Therefore, Re affects the mixing efficiency, and the higher the Re, the higher the mixing efficiency, and the shorter the micromixer channel length required for complete mixing. In the absence of turbulence flow, mixing fluids of large diameters of molecular substances, the diffusivities of which are in the order of 10^−10^ m^2^s^−1^ or less, mostly depended on passive molecular diffusion which is a slower process than convection. Simple straight channels of T or Y junctions of micromixers would need tens of centimeters of mixing length to obtain complete mixing [18], which is impractical for most integrated microfluidic systems. Hence, effectively achieving complete mixing performance in short microchannel lengths is a major challenge in this field.

In the past decade, magnetic force [19,20], electrical force [21,22], piezoelectrical force [23], and Lorentz force [24] have been the main power inputs to enhance mixing efficiency in an active micromixer. Although active micromixers show their potential ability for a rapid mixing process, the fabrication and integration of the active components into a microfluidic system are difficult and inconvenient as well as expensive for commercial production. Taking a micromixer which utilizes piezoelectrical force as an example [25], it needs not only fabrication of the embedded piezoelectric actuator, but also wiring of the piezoelectric actuator to an internal power supply. In contrast to active micromixers, passive micromixers solely depend on specially designed geometries to enhance the efficiency of passive diffusion through fluid bending, twisting, stretching, folding, overlapping, or splitting recombination to reduce mixing time and length. Passive micromixers are advantageous over active micromixers owing to their lower cost, simpler design, easier integration, and convenience for mass production.

Currently, various kinds of passive micromixers are classified by their structure and mixing principle. According to their structure, the passive micromixers are divided into two categories: the planar micromixers and three-dimensional (3D) multilayer micromixers. In addition, lamination micromixers and chaotic advection micromixers are recognized as the two main types of mixer based on the mixing principle. Compared with planar micromixers, the 3D micromixers are normally complicated in terms of fabrication, however, they can more effectively induce vortex of flow and 3D reactive flows, having fundamentally novel dynamical features not found in 2D systems [26,27,28,29,30,31,32,33,34,35,36]. Both lamination and chaotic advection micromixers rely on elongating fluid streams’ interfacial areas to obtain better mixing quality. However, the lamination micromixers usually depend on splitting and recombination of flow streams, and the chaotic advection micromixers normally rely on vortex or secondary fluid flow to distort, rotate, stretch, and fold flow on the cross-section to achieve better mixing performance. Therefore, the chaotic advection micromixer has attracted increasing attention recently.

In this study, we developed a three-dimensional passive micromixer with embedded oblique ridges at the bottom and top of the microchannel. This has the advantage of easy fabrication and quickly achieves high mixing efficiency over a short distance. In order to clearly understand the characteristics of fluid flow in the microchannel, numerical simulation of the micromixer was carried out. Different parameters, such as fluid properties and Re number, were studied to investigate their effects on the mixing performance of this micromixer. An experiment was also carried out to measure the fluid mixing performance in this micromixer. Compared with previously published works [37], the novelty of this work is that we combined the oblique ridge design and the split and recombine mixer design into one 3D structure. This unique structure can effectively improve the efficiency of the micromixer. Thus, the micromixer can quickly and completely mix the fluid through a short microchannel length. It is expected that the micromixer will be used as a fluid mixing module in microfluidic micromachines and applied in fields such as pathogen detection to reduce the overall size of the system.

## 2. Materials and Methods

### 2.1. Micromixer Design

**Figure 1:**
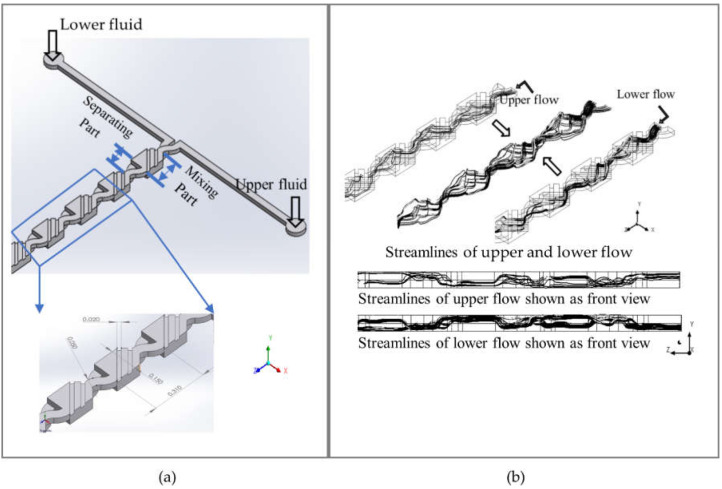
(**a**) Structure and schematic diagram of the micromixer, the dimensions are in millimeters; (**b**) schematic diagram of design concept with streamlines of fluids in the micromixer.

A 3D structure of the micromixer is illustrated schematically in Figure 1a. There are two layers for injecting upper and lower flow in the micromixer structure, including ten cells. Each cell contains two parts. One part is used for fluid mixing and the other part for fluid separation. The widths of the inlet and oblique ridges are 0.050 mm and 0.02 mm, respectively, and the depth is 0.025 mm. The length of the whole cell is 0.310 mm.

Two fluids are injected separately and synchronously into the upper and the lower layers. In the mixing part, the fluids are expected to transport and distort from one layer to another via the embedded ridges, which can induce transverse motion of fluids. As shown in Figure 1b, the streamlines of the upper layer or the lower layer only are illustrated to easily observe fluid flowing in the micromixer. On the base of the longitudinal symmetric structure, the statuses of streamlines in both layers are obviously similar to each other. Fluid flows in the mixing part are not only distorted and rotated when passing through the upper and lower layer, but are also expanded to a wider flow. These reoriented streams re-stretch the merged part of the fluids. In the separating part, the fluids are expected to compress and divide into two sub-flows in the upper and lower layers. The bended separating channel can continue to rotate sub-flows. Then, these two sub-flows flow into the mixing part to begin the next mixing loop.

**Figure 2:**
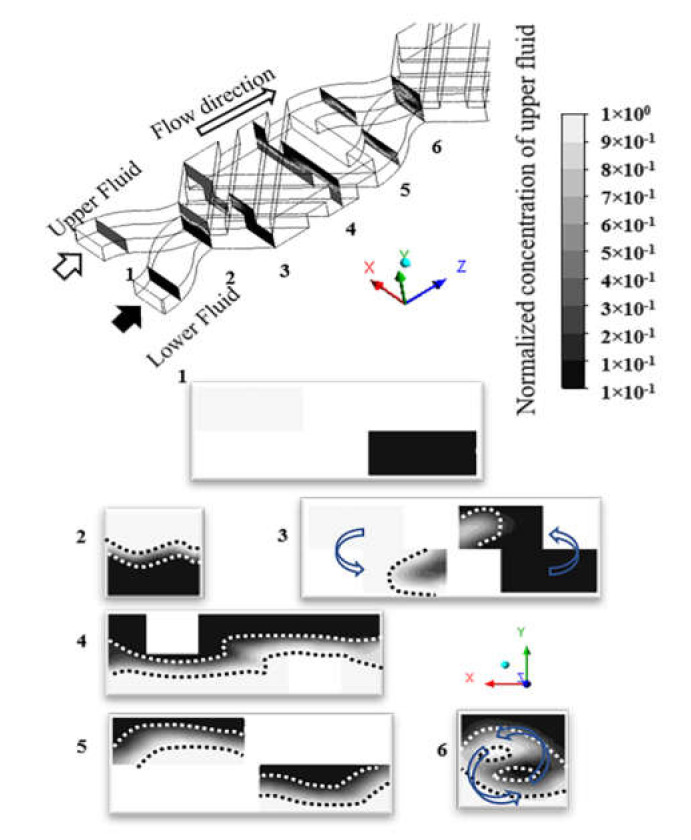
Schematic diagram of design concept with procedures of fluid mixing illustrated through mixing patterns.

The multi-step mixing procedure in the micromixer is shown in Figure 2. In the color map, 1 and 0 represent two pure fluids for the upper and the lower layer inlets, respectively, as shown in cross-section ‘1’. After passing the separation part, a waving contact interface is shown on the cross-section ‘2’, marked by the black and white dashed lines. The fluid in the region between the dashed lines is the mixed fluid. It shows that the upper fluid is transferred to the lower layer and the lower fluid is transferred to the upper layer counterclockwise. Meanwhile, the region of mixed fluid is distorted and expanded to a larger area, as shown in sections ‘3’ and ‘4’. The cross-section ‘5’ in the second cell shows the mixed fluids in the upper and lower layers. Cross-section ‘6’ in the second cell shows the region of mixed fluids is larger than the one in cross-section ‘2’ in the first cell. The region of mixed fluid continues to expand through several cells until all fluids are completely mixed.

The exponential and varied shape of the enlargement interface verifies that it is induced by the rotating flow and it therefore can generate chaotic advection [18,35]. Hence, the fluids can obtain thorough mixing when relying on repeated sequences of converging and splitting by passing through several cells. Most importantly, the generating transverse flow as part of 3D rotation flow (also called the secondary flow) could produce a vortex to stir the fluids rapidly to induce chaotic advection, which can efficiently boost fluid mixing.

### 2.2. Numerical Modeling

The fluids in the micromixer are assumed to be Newtonian fluids. The body force is ignored by assuming the fluids are steady and incompressible. Based on these assumptions, the governing equations include a continuity equation, Navier–Stokes equation, and species convection–diffusion equation which can be described as:

(1) ∇·V=0

(2) $$\rho V\cdot \nabla V=-\nabla P+\mu \nabla^{2}V$$

(3) $$V\cdot \nabla C=D\nabla^{2}C$$

where V is fluid velocity vector, ρ is the fluid density, P is the pressure, μ is the fluid dynamic viscosity, C is the species mass concentration, and D is the diffusion coefficient of the species.

ANSYS Fluent is employed to solve Equations (1)–(3). In the Fluent solver, we choose pressure-based and steady-state laminar flow without reaction mixing. Energy and species transport models are selected, and two fluid concentrations are set as 1 m/mol and 0 m/mol, respectively. For the boundary conditions, all inlet velocities are set as constant; outlet pressure is set as static pressure of 0 Pa; no slip shear conditions are set for all channel walls. SIMPLIC scheme is used for coupling of pressure and velocity.

The representative normal fluid and hard-to-mix fluid are used to investigate their effects on the mixing performance. Densities of both fluids are set as 997 kg/m^3^. The normal fluid, with a viscosity of 9.7 × 10^−4^ kg/ms and diffusion coefficient of 3.6 × 10^−10^ m^2^/s, is easier to mix, and those parameters are normally adopted for numerical simulation [17,20,24,25]. The hard-to-mix fluid with a high viscosity and low diffusion coefficient of 0.186 kg/ms and 9 × 10^−13^ m^2^/s is also simulated to verify our micromixer’s ability to mix high-viscosity fluid. A series of Re numbers, as listed in Table 1, is used to investigate mixing performance of the micromixer, where Re numbers are varied from 0.007 to 66.7 for the normal fluid and from 0.0007 to 0.7 for the hard-to-mix fluid.

A high Re number may enhance mixing efficiency, but the corresponding inlet velocity requires a relatively high pressure. For example, in Wong et al.’s paper [17], when 1 bar pressure is applied, the Re number can reach 120 at an inlet velocity of 1.8 m/s. At this high pressure, the micromixer demands a special function of the instrument and better bonding and material strength. PDMS cannot endure such high pressure. Thus, using high velocity to achieve high mixing efficiency is impractical. However, velocities of 1.946 m/s (Re number 66.7) for normal fluid and 3.73 m/s (Re number 0.7) for hard-to-mix fluid, which requires high pressure, are still used in our numerical simulation for comparison purposes.

A mixing index to measure the mixing performance is described by flowing equations:

(4) $$\sigma =\sqrt{\frac{1}{N}\overset{N}{\underset{i=1}{\sum }}{\left(C_{i}-C_{\infty }\right)}^{2}}$$

(5) $$\sigma_{max}=\sqrt{\frac{1}{N}\overset{N}{\underset{i=1}{\sum }}{\left(C_{0}-C_{\infty }\right)}^{2}}$$

(6) $$M=1-\frac{\sigma }{\sigma_{max}}$$

where σ is the standard deviation of the concentration of the cross-sectional plane, *N* is the total number of samples in the cross-section, $C_{i}$ is the mass fraction of the number *i* sample, $C_{\infty }$ is optimal mixing mass fraction and its number should be 0.5 when two fluid species of the same amount are well mixed, $\sigma_{max}$ is the maximum of f σ, $C_{0}$ is the original mass fraction that should be 1 or 0, and *M* is the mixing index, with a range from 0 for no mixing to 1 for complete mixing. Normally, a mixing index of 0.9 can be considered as complete mixing.

### 2.3. Micromixer Fabrication and Experiment Setup

**Figure 3:**
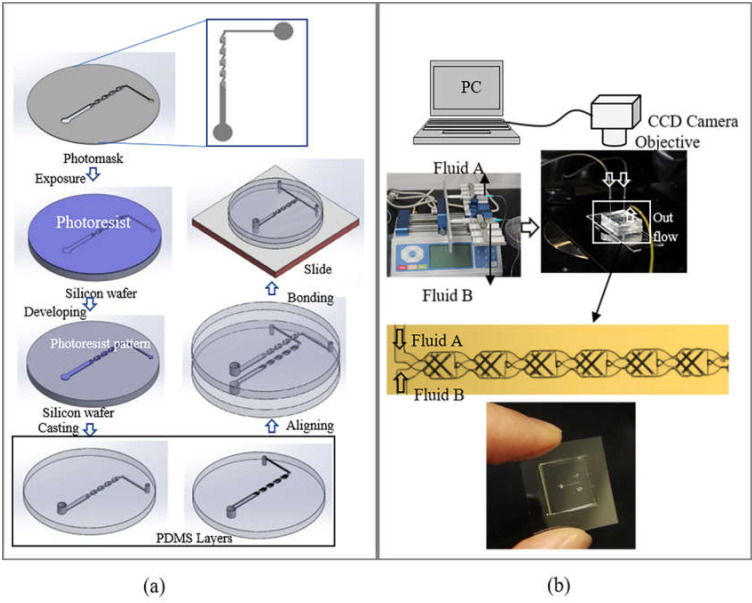
(**a**) Fabrication procedure of the micromixer using PDMS; (**b**) experiment setup.

Fabrication procedures of the micromixer are illustrated in Figure 3a.

A micromixer is fabricated by using the standard soft lithography (also called replica modeling) method, in which upper and lower layers of the micromixer use the same photomask pattern. The ridges and channel can be modeled in the same process, because they are designed to be of the same depth. A clean two-inch silicon wafer is initially dehydrated at 95 °C for 2 min, and then it is immediately placed into a desiccator for cooling. The cleaned and dried silicon wafer is coated with a 25 μm thick film of SU-8 2025 (SU-8) negative photoresist by using a spin coater at 3000 rpm for 1 min. Before and after exposure to UV light with a wavelength of 360 nm and energy of 240 mJ cm^−2^, the coated silicon wafer is prebaked (65 °C for 3 min) and postbaked (95 °C for 6 min and 65 °C for 3 min). In the next step, the wafer is washed with developer (1,2-propanediol monomethyl ether acetate) to remove unexposed SU-8, then it is rinsed by isopropyl alcohol to form the SU-8 pattern. Next, polydimethylsiloxane (PDMS), a mixture of the silicon elastomer (A) and a curing agent (B), is used to make the microchannel structures. Hardness of the structures can be controlled by using different ratios of A and B. We adopt ratios of 5A:1B and 10A:1B by weight to make two microchannel layers, respectively. Well-mixed PDMS solution is poured onto the pattern silicon wafer, and then immediately put into a vacuum to remove air bubbles created during the mixing process. After curing in an oven at 80 °C for 1 h, the PDMS replica is peeled off from the pattern silicon wafer. Subsequently, holes are punched into the PDMS slab to create an inlet and outlet. A microscope is used to align two PDMS replicas made by PDMS mixtures with different ratios. Two well-aligned replicas are pressed to remove air and placed in an oven at 80 °C for 1 h. Meanwhile, a clean glass slide is spun at 2000 rpm for 75 s to coat with thin PDMS with a 20A:1B ratio. Then, the coated glass slide is placed in an oven at 80 °C to cure for 1 h. Finally, the well-aligned replicas and glass slide are pressed together for bonding and placed into an oven at 80 °C overnight.

## 3. Results

### 3.1. Results and Discussion of Numerical Simulation

**Figure 4:**
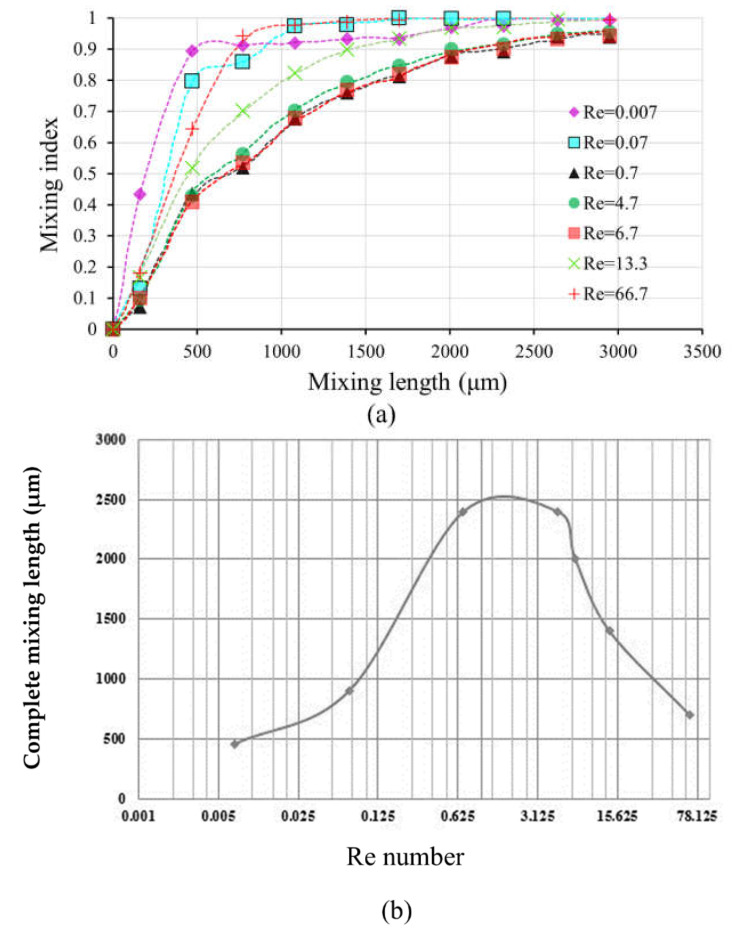
(**a**) The mixing index of each cross-sectional plane in the micromixer at different Re numbers for the normal fluid; (**b**) complete mixing lengths versus Re number in the micromixer for the normal fluid.

To quantify the mixing performance, the mixing index is calculated for each cross-sectional plane which is located at the joint of the separating and mixing parts. An accurate mixing index can be obtained when the cross-sectional plane is meshed by about 1500 cells. For the normal fluid, seven curves of the mixing index versus mixing length for different Re numbers are shown in Figure 4a. In addition, the relationship between complete mixing length and Re number is characterized and shown in Figure 4b, based on the information obtained from Figure 4a, where the mixing length has a mixing index of 0.9 and is denoted as complete mixing length.

Figure 4b shows that the shortest complete mixing length of around 500 µm occurs at the Re number of 0.007. The complete mixing length increases to about 2400 µm as the Re number increases from 0.007 to 4.7. However, with further increasing of the Re number from 4.7 to 13.33, the complete mixing length decreases to about 1400 µm, and consequently decreases to about 650 µm as the Re number increases to 66.7. Based on this result, a critical Re number of 4.7 is obtained for this micromixer. When the fluids in the micromixer flow at Re numbers lower than the critical value, the mixing process mainly relies on the mechanism of splitting–recombination. In this case, as the fluids flow slowly at the lowest Re number (0.007), even though a complete mixing is achieved at a short mixing length of 500 µm, it takes a long period of time to complete the mixing. Therefore, fluids mixing at low Re numbers are not acceptable for the application of rapid testing. As the Re number increases and passes the critical value, the fluid mixing is dominated by strong advection which effectively boosts mixing performance. In this case, a shorter complete mixing length can be obtained at higher Re numbers. Even though the complete mixing length may be further decreased by increasing the Re number, there is a limitation on the Re number for each micromixer design.

**Figure 5:**
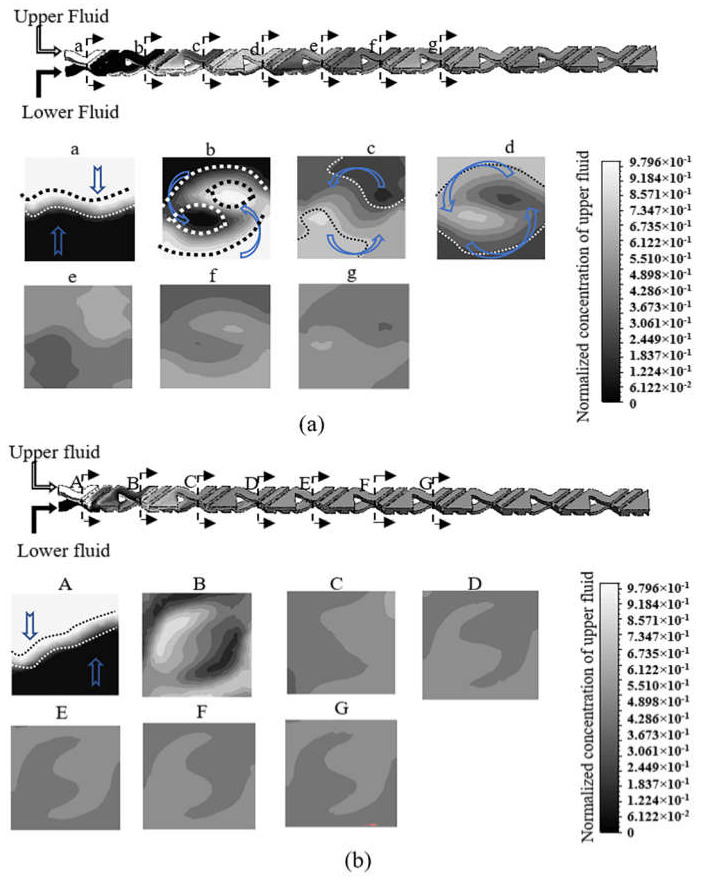
The mass fraction contours of cross-section planes from simulation result at (**a**) Re number of 4.7 and (**b**) Re number of 66.7 for the normal fluid (mesh size 10 μm).

For further understanding of the previous results, mass fraction contours of cross-section planes at Re numbers of 4.7 and 66.7, as shown in Figure 5, are used to investigate the fluid mixing phenomena in the micromixer. At the cross-section ‘a’ in Figure 5a, two fluids conflate and overlap vertically to form a small fluid mixing region between two dashed lines. After flowing through one cell, the cross-section ‘b’ showed that the fluid mixing region increases rapidly and the mass of fluids is transferred to opposite sides by counterclockwise flow. The fluid mixing region continues to expand and distort and the counterclockwise motion of flow indicates the existence of 3D rotating flow, which is proved to have the capability to fold, stretch, and distort fluid to generate large regions of contact interface [36,38]. Accompanying the fluids continuing to pass through the designed microchannel structure, separation, recombination, and rotation repeatedly affect the fluid mixing. Therefore, at cross-section ‘e’ in the fifth cell, the fluid mixing region is extended to occupy the whole cross-section. The contour at cross-section ‘e’ to ‘g’ shows that the completely mixed fluids increase from cross-section ‘e’ to ‘g’, where all the fluids in the cross-section ‘g’ are almost mixed completely.

The different contours at the Re number of 66.7 is illustrated in Figure 5b. At the cross-section ‘A’, a small fluid mixing region is formed between two dashed lines in the first cell. However, the fluid mixing region exponentially increases to occupy the whole cross-section ‘B’ in the second cell. Then, complete mixing is quickly achieved at the cross-section ‘C’ in the third cell. The results indicate that chaotic flow is generated due to strong advection.

**Figure 6:**
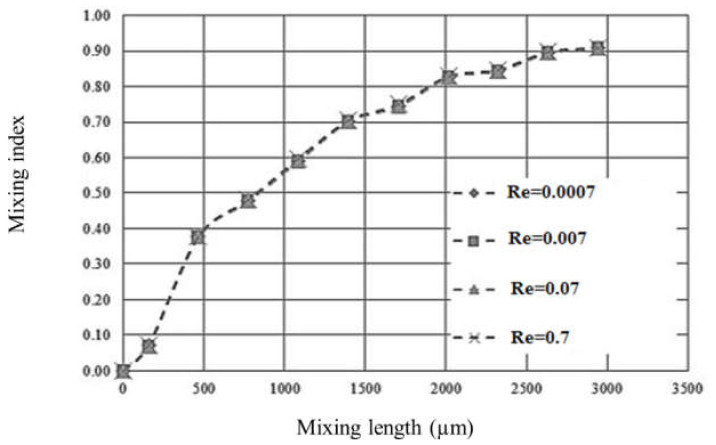
The various mixing indexes of cross-sectional plane versus mixing length at different Re numbers for the hard-to-mix-fluid.

The mixing performance of hard-to-mix fluids is shown in Figure 6, where curves of mixing indexes versus mixing length are identical for different Re numbers ranging from 0.0007 to 0.7. The results also show that the complete mixing length is not changed as the Re number increases from 0.0007 to 0.7. It implies that no chaotic flow occurs for the mixing of hard-to-mix fluid. However, our micromixer can still achieve a complete mixing length of about 2550 µm.

### 3.2. Experimental Results and Discussion

During the experiment, phenolphthalein and sodium hydroxide (NaOH) are used as testing fluids. NaOH in DI water is a basic solution, and the color is transparent. As a pH indicator, 0.2% phenolphthalein solution in 99% ethanol will change color from transparent to pink when the pH is higher than 7 by reacting with 0.2 M NaOH solution. Mixing of dye liquors is another common method to evaluate the mixing performance. Purple and green food dyes (produce by ACH Food Company) are also used as testing reagents to observe the mixing performance.

**Figure 7:**
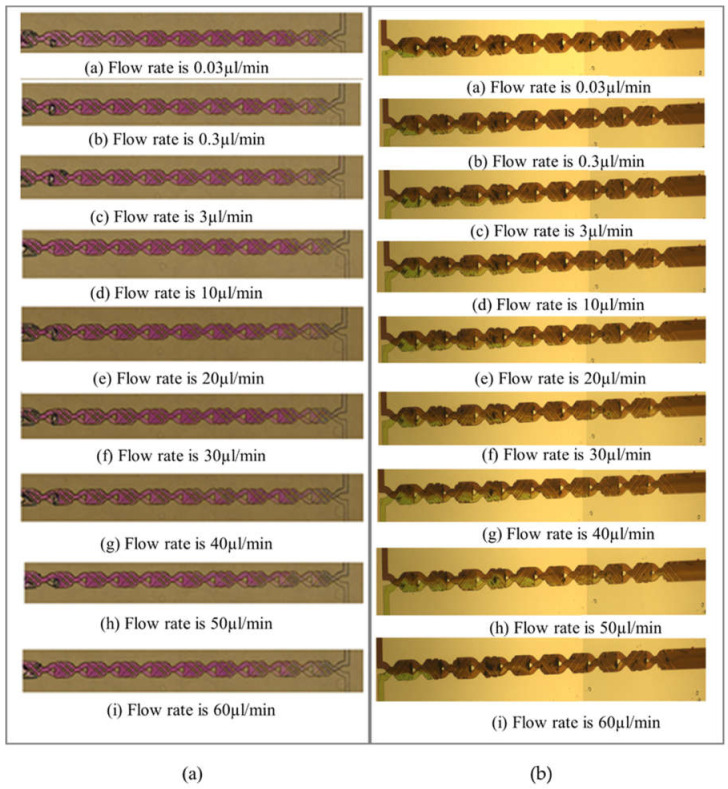
Experimental results of mixing performance of (**a**) phenolphthalein-NaOH and (**b**) purple–green dye at various flow rates.

The mixing performance of phenolphthalein and NaOH under a flow rate from 0.03 µL/min and 60 µL/min is presented in Figure 7a. At the front of the first cell, almost no mixing happens between the two solutions, hence the color is still colorless or transparent. Passing through two cells (620 µm) or fewer, light pink begins to appear, and it gradually changes from light pink to dark pink. Normally, there is no further color change after the third cell, which indicates that the two solutions are completely mixed. Based on these experimental results, the designed micromixer is much more efficient than most passive micromixers, which normally need several millimeters [16,17,22].

As shown in Figure 7b, the green and dark purple dyes both fill the first cell at various flow rates, indicating the two fluids are not mixed in the first cell. Then, the green color gradually turns into purple after passing through several cells. Note that the purple color does not show an obvious color change when the green fluid flows into it; thus, the mixing efficiency is evaluated by counting the area that is dominated by purple. When the green color completely vanishes, complete mixing is considered to be achieved. At a flow rate of 0.03 µL/min, the green color disappears, and complete mixing is obtained after passing fewer than two cells (around 500 µm). However, the mixing performance worsens and three to eight cells (around 930 µm to 2400 µm) are required to achieve complete mixing when inlet velocities increase from 0.3 µL/min to 50 µL/min. At a flow rate of 60 µL/min, the fluids achieve complete mixing after only passing through two cells (around 600 µm).

**Figure 8:**
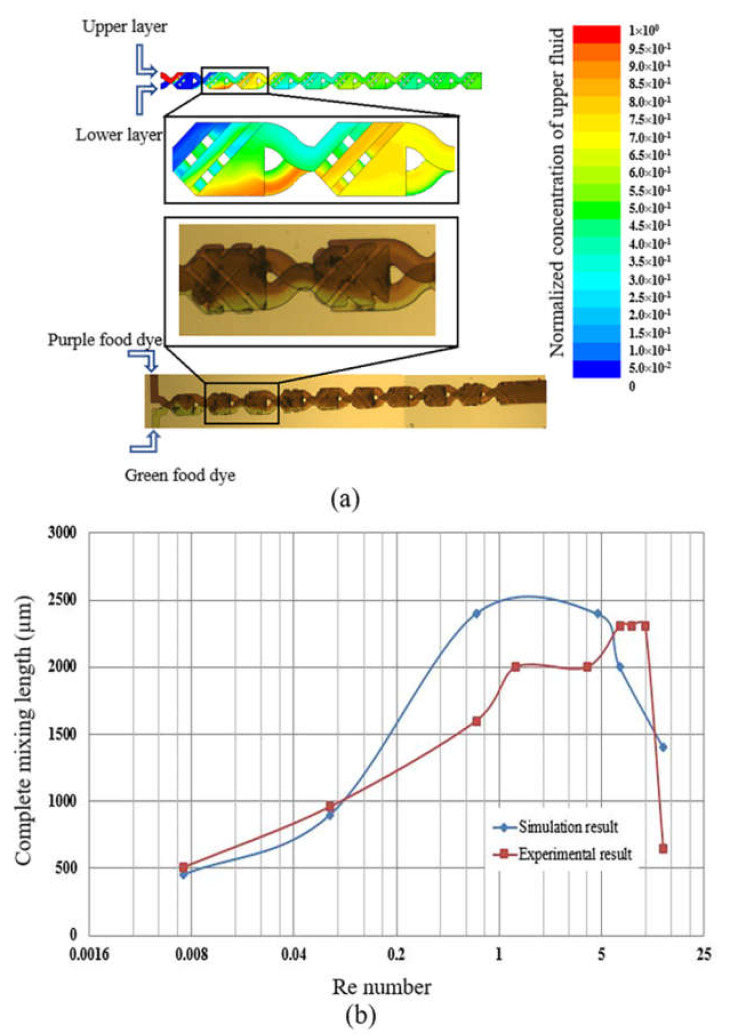
(**a**) Comparison of experimental and simulation results at a flow rate of 3 µL/min; (**b**) Comparison the trend of curve of complete mixing lengths between experimental and simulation results in the micromixer at variation of Re numbers.

We compare the experimental and simulation results for the Re number of 0.7 which relates to a flow rate of 3 µm/min. The contour of the mass fraction from the simulation is shown in Figure 8a, where the pure fluids in the upper and lower layers are represented by red and blue in the first cell, respectively. The results show that the mixing performance from experiments and the simulation are similar, where complete mixing is achieved after the 4th cell.

The curves of the complete mixing lengths versus Re number for the experiment with food dyes and the simulation are also compared and shown in Figure 8b. Both curves show a similar trend, where they climb upwards when the Re number is around 0.007 (flow rate of 0.03 µm/min) to around 10 (flow rate of 50 µm/min), then descend when the Re number is around 13.3 (flow rate of 60 µm/min). Due to the limitation of the simulation parameters, the simulation only shows the best results in a near-ideal state. However, the experiment is affected by various factors, such as microfluidic chip fabrication, the temperature, the operations, etc., making the real experimental results unable to achieve the ideal simulation state. Therefore, one can conclude that the results of numerical simulation are fairly consistent with experimental results in terms of trends.

A novel three-dimensional passive micromixer has been successfully developed, fabricated, and analyzed through numerical simulation and experiments. Based on the simulation results, a critical Re number is obtained, which is 4.7 for this designed micromixer. When the Re number is smaller than the critical value, the fluid mixing is dominated by the mechanism of splitting–recombination, otherwise it is dominated by chaotic advection. A complete mixing length of 500 µm can be obtained at a very small Re number of 0.007 and increases to 2400 µm as the Re number increases to the critical value of 4.7. These results showed that a shorter complete mixing length can be obtained at a lower flow rate. However, mixing at a lower flow rate is not practical in microfluidic applications, where a test needs to be completed in a very short time. As the Re number keeps increasing and passes the critical value of 4.7, the complete mixing length continues to descend to 650 µm at the Re number of 66.7, which indicates that a short complete mixing length can be obtained at a higher flow rate. For the hard-to-mix fluid, even though no evidence of strong chaotic advection is presented in the simulation, the micromixer can still achieve a complete mixing length of 2550 µm. Hence, our proposed micromixer can effectively improve the mixing process; furthermore, it can be easily integrated into other microfluidic micromachines through conventional microfabrication technology and be used as a module in pathogen detection and other fields to reduce the volume of the entire system.

## Figures and Tables

**Table 1:** Re numbers for the micromixer.

Normal Fluid	Hard-to-Mix Fluid
Re	
0.007	0.0007
0.07	0.007
0.7	0.07
4.7	0.7
6.7	
13.3	
66.7	
